# Effects of seed infection by *Fusarium verticillioides* on maize performance against *Sesamia nonagrioides* attack

**DOI:** 10.1111/ppl.14649

**Published:** 2024-12-03

**Authors:** N. Gesteiro, A. Cao, R. Santiago, P. Lobagueira, S. J. González‐Prieto, R. A. Malvar, A. Butrón

**Affiliations:** ^1^ Misión Biológica de Galicia, Sede de Pontevedra (CSIC) Pontevedra Spain; ^2^ Agrobiología Ambiental, Calidad de Suelos y Plantas (UVIGO), Unidad Asociada a la MBG (CSIC) Spain; ^3^ Misión Biológica de Galicia, Sede de Santiago (CSIC) Santiago de Compostela Spain

## Abstract

In maize (*Zea mays* L), the fungus *Fusarium verticillioides* can behave as a pathogen, but it is also able of asymptomatic colonization as an endophyte. Therefore, it would be of great value to identify metabolites and/or metabolic pathways implicated in mutualistic and pathogenic interactions. The objectives of the present study were: (i) to investigate the effect of seed colonization by *F. verticillioides* on maize growth in a group of inbreds with contrasting resistance to *F. verticillioides*; (ii) to know if maize priming by *Fusarium* seed infection affects maize response to other parasites and if these differences could depend on genotype resistance to *Fusarium*; and (iii) to determine which metabolites could be associated to beneficial/detrimental changes on maize performance. Targeted and untargeted metabolomic approaches were carried out to characterize the response of control and primed plants to the most common maize pest in the Mediterranean area, *Sesamia nonagrioides* Lefèbvre (Lepidoptera: Noctuidae). The study cannot assume a differential pattern of infection between resistant and susceptible inbreds, but seed inoculation with *F. verticillioides* upon infestation with *S. nonagrioides,* significantly altered defense metabolism in resistant inbreds. Meanwhile it also induced a lipid response in susceptible inbreds that could mediate their increased plant susceptibility to insect attack. Although an endophytic interaction between the fungus and specific genotypes cannot be proven, defense pathways were favorably altered by *F. verticillioides* colonization among resistant inbreds.

## 1 INTRODUCTION

Manipulating biotic interactions between plants and microorganisms to provide desired nutritional or defensive advantages to the plant and thus reduce or eliminate the need for external inputs such as fertilizers and/or pesticides is fundamental to the practice of ecologically sound agriculture. The challenge is how to encourage positive interactions while reducing negative interactions. In maize, some environmental conditions determined by the climate and maize genotype are conducive to Fusarium ear rot (FER) disease and kernel fumonisin contamination caused by *Fusarium verticillioides*, but this fungus is also able of asymptomatic colonization as an endophyte (Bacon et al., 2008; Santiago et al., 2015). Endophytes are microbes like fungi or bacteria, that can colonize the internal plant tissues without causing any obvious damage or disease symptoms. Instead, these microorganisms could be beneficial for plant growth and health (Ali et al., 2017). In this sense, *F. verticillioides* has already been proven as a maize endophyte due to its contribution to host fitness through growth promotion, induction of defense‐associated changes such as lignin deposition and interference of infection by other fungi (Yates et al., 1997, Yates et al., 2005; Lee et al., 2009; Rodríguez Estrada et al., 2012; Blacutt et al., 2018). However, the dual nature of *F. verticillioides* as both pathogen and endophyte indicates a complex relationship with maize in which interactive biotic factors such as maize genotype and abiotic factors may alter the required balanced relationships, resulting in a weakened plant and changing the relationship into a disease, during which mostly mycotoxins fumonisins are produced (Bacon and Hinton 1996; Bacon et al., 2008). Understanding this complex relationship and the maize genotypic factors that could be determinant for favoring/hindering fungal endophytic behavior would help in selecting breeding targets for resistance to FER and kernel contamination with fumonisins.

Previous studies have tried to elucidate the role of the presence of *F. verticillioides* on the aggressiveness of the well‐known maize pathogen *Ustilago maydis as both biotic stressors* have long been associated with their host plant maize, and co‐occur frequently (Lee et al., 2009; Rodríguez Estrada et al., 2012). These studies concluded that growth of the pathogen *U. maydis* on maize was lower when the endophyte *F. verticillioides* was present, probably due to antagonistic interactions between the endophyte and the pathogen. However, *F. verticillioides* may facilitate *U. maydis* infection because the endophyte benefits from the interaction with *U. maydis*. The maintenance of defensive mutualism will depend on interactions of the parasite with the host as well as with the defensive symbiont because, when the endophyte *F. verticillioides* was inoculated after *U. maydis*, increased disease severity caused by *U. maydis* was observed as well as evidence of *F.verticillioides* behaving as a pathogen. Yet, co‐inoculation of both fungi significantly decreased smut disease and plant growth was increased (Lee, Pan et al., 200; May and Nelson 2014).

*Fusarium verticillioides* is often found inside maize kernels in temperate areas around the world and can be seed‐transmitted to the next generation, symptomless systemic infection of the growing plant being a common fact (Wilke et al., 2007; Santiago et al., 2020). In the Mediterranean area, as common as maize seed colonization by *F. verticillioides*, is the stem tunneling by the Mediterranean corn borer, *Sesamia nonagrioides* Lefèbvre (Lepidoptera: Noctuidae, Velasco et al., 2007; Kacar et al., 2023). As far as we know, there is no work on the possible beneficial or detrimental effect of kernel colonization by *F. verticillioides* on maize performance against pest attacks. Therefore, in the current study, we have explored the possible mutualism between the fungus and the plant focusing on plant growth but also in plant defense against a well‐known biotic stress factor, the attack by stem borers. Fungal endophytes gain shelter, nutrition, and dissemination via host seeds, and can contribute to an array of host fitness enhancements including protection against insects and other fungi (Schardl et al., 2004). According to the observation that resistance of sugarcane cultivars (*Saccharum sp*.) to the stem borer *Eldana saccharina* was conditioned by sugarcane stalk colonization by *Fusarium* species, we hypothesized that maize seed infection by *F. verticillioides* could also interfere on maize resistance to the stem borer attack (Mahlanza et al., 2014). Therefore, un‐targeted coupled with targeted metabolic approaches were applied to uncover biomarkers and to check if known metabolites are associated to insect resistance and would be affected by seed infection with *F. verticilllioides*. Altering the plant nutritional value is a common defense mechanism of plants against insect attacks and, more specifically, the maize cell wall has been proven as a biochemical‐structural barrier against corn borer attacks (Buendgen et al., 1990; Bergvinson et al., 1997; Ostrander and Coors 1997; Awmack and Leather 2002). Several studies have pointed out that lignin quantity and composition as well as hydroxycinnamate content are the most determinant cell wall components for stem borer resistance (Santiago et al., 2006; Barros‐Rios et al., 2015; Gesteiro et al., 2021; Lopez‐Malvar et al., 2021).

Overall, the objectives of the present study were: (i) to investigate the effect of seed colonization by *F. verticillioides* on maize growth in a group of maize inbreds with contrasting resistance to *F. verticillioides*; (ii) to know if maize priming by *Fusarium* seed infection affects maize response to other parasites and if response differences could depend on genotype resistance to *Fusarium*; and (iii) to determine which metabolites could be associated to beneficial or detrimental changes on maize performance.

## 2 MATERIALS AND METHODS

### 2.1 Experimental design

The eight inbred lines used in this study and their performances under different biotic factors are shown in Table 1. Four different treatments were applied to the eight inbred lines (Table 1): Seed inoculation with *F. verticillioides* and no infestation with *S. nonagrioides* (FC), seed inoculation with *F. verticillioides* and infestation with *S. nonagrioides* (FS), no seed inoculation with *F. verticillioides* and no infestation with *S. nonagrioides* (CC) and no seed inoculation with *F. verticillioides* and infestation with *S. nonagrioides* (CS).

**TABLE 1:** Inbred lines and their levels of resistance against Fusarium ear rot (FER).

Inbreds	Pedigree	FER
**A239**	A347 × A73	Susceptible
**A509**	A78 × A109	Resistant
**A630**	(A116 × WF9) WF9^3^	Resistant
**A637**	CO106 × A321	Resistant
**EP125**	Selection of CO125	Susceptible
**EP42**	Tomiño	Susceptible
**EP77**	EP31 × CM109	Resistant
**PB130**	Rojo Vinoso de Aragón	Susceptible

Classification based on data recorded by Santiago et al., (2013).

Eighty seeds from each inbred line were surface and internally disinfected (by heat exposure after being imbibed in water for 4 h) to eliminate seed‐borne fungi using procedures developed by Daniels (1983) and modified by Yates et al., (1997). Specifically, kernels placed in sterilized plastic cups were covered with 5.25% sodium hypochlorite, agitated vigorously for 10 min, rinsed twice with sterile distilled water, imbibed in fresh water for 4 h at 25°C, heat‐treated for 5 min at 60°C, and rinsed in cool water. Non‐inoculated control seeds (40 from each inbred line) were sown directly. The remaining seeds were submerged in fungal inoculum (10^6^ spores ml^−1^) for 3 min at room temperature before being sowed. Forty seeds from each genotype‐inoculation treatment combination (seed inoculation with *F. verticillioides* or no inoculation) were sown in 16 pots (2–3 seeds per pot). FC treatment on the inbred A637 was lost due to germination problems. Later, when the plants were at five‐six leaf stages (V5‐V6), one plant per pot was left and half of the pots of each genotype‐inoculation treatment combination were placed in the corresponding treatment sector of the greenhouse. The two treatment sectors were perfectly isolated from each other with meshes to prevent the larvae pass from one to another in the following steps. Before the tasseling stage, all the plants in one greenhouse sector, eight for each genotype‐inoculation treatment combination, were infested with two *S. nonagrioides* larvae per plant. Approximately 30 days later, when all the plants were already at the reproductive state, plant height was measured and stems were split longitudinally and the tunnels made by *S. nonagrioides* larvae were assessed in each individual plant, six for each genotype‐inoculation treatment combination. No damage by *S. nonagrioides* larvae was detected among non‐infested plants, but some plants were undamaged in the infestation treatment and were discarded. Therefore, six replicates were used for each genotype‐inoculation treatment combination, except for A239, A509 and EP42 under CS and FS treatments (five for each genotype‐inoculation treatment combination) and A637 under FC treatment (none) and, consequently, data were available for 180 plants. As the stem pith is the maize tissue chosen by borers for feeding purposes, stem pith samples were taken from the lower half of the first internode below the main ear of six individual plants per genotype‐treatment to perform un‐targeted metabolomic and nutritional analysis and the upper half was used for quantification of cell‐wall components. Pith samples were frozen at −80°C, lyophilized and ground.

### 2.2 Untargeted metabolomic analysis: extraction, MS acquisition and processing of MS spectra

Twenty milligrams of stem pith tissue per sample were extracted with 1 mL of 80% methanol, mixed in a vortex, sonicated in an ultrasonic bath for 15 min, and centrifuged at 20 000 *g* for 15 min. The supernatant was filtered through a 0.22 μm PTFE membrane to an Eppendorf tube and an aliquot was transferred to a HPLC certified vial. Sample pools were prepared in separate vials for MS/MS analysis, and balanced pools of the total sample extracts were prepared as quality control (QC). Blank extractions were used for capturing contaminations introduced during the extraction process. All samples were evaporated to dryness, stored at 4°C until analysis, and then dissolved in 80% methanol.

Metabolomics profiles of methanolic extracts of the pith samples were acquired using an ultra‐high‐performance liquid chromatography (UHPLC) system (Thermo Dionex Ultimate 3000 LC) coupled to a quadrupole‐time‐of‐flight mass spectrometer (QTOF‐MS) equipped with an electrospray ionization source (ESI; Bruker Compact; Bruker Daltonics).

We measured the analytical samples using three analytical batches, each one containing six blocks of 10 analytical samples. The analytical samples were randomly assigned to batches and blocks within batches. Blanks were placed at the initial and final positions of each batch and QC samples were evenly distributed bordering the blocks. UHPLC separation was performed with a Intensity Solo 2 C18 column (1.7 μm, 2.1× 100 mm; Bruker Daltonics) in a gradient elution that consisted of 0.1% of formic acid on water (solvent A) and acetonitrile (solvent B) as mobile phase in a 0.4 mL min^−1^ flow rate. The elution conditions were: 0 min, 3% B; 4 min, 3% B; 16 min, 25% B; 25 min, 80% B; 30 min, 100% B; 32 min, 100% B and the return to initial conditions was at 33 min (3% B) for 3 min.

Full scan MS data were acquired in both positive and negative ionization modes over the mass range of 100–1200 m/z, and under the following specific conditions: gas flow 9 L min^−1^; nebuliser pressure 2.6 bar; dry gas 9 L min^−1^; dry temperature of 220 °C. Auto MS/MS fragmentation was performed in pooled samples to facilitate compound identification. After each batch, the MS ion source was cleaned, and the MS was recalibrated.

We pre‐processed the raw MS spectra using the algorithm T‐Rex 3D in MetaboScape 4.0 software (Bruker Daltoniks). Parameters were set to separate measured peaks from background noise and features were grouped across samples and corrected for retention time shifts. After this pre‐processing, data were prepared for statistical analysis using Metaboanalyst (Chong et al., 2019). Features with >75% missing data were eliminated and missing data imputation was performed using the KNN (feature‐wise) method that estimates missing values in a dataset by considering the values of the closest data points, determined by a distance metric like Euclidean distance. The missing value is then assigned the average of these nearest neighbors' values, weighted by their proximity. Afterwards the ANCOVA method was used for batch correction and contaminants present in blanks were removed. Features with percent relative standard deviation (RSD = SD/mean) > 25% across QCs samples were removed, as well as uninformative features that presented near‐constant values detected by the interquartile range filter (IQR). Then, Pareto scaling was applied to adjust for the disparities in fold differences between the analytes.

### 2.3 Effect of seed infection by *F. verticillioides* on the defenses and nutritional value of the maize stem pith under infestation with *S. nonagrioides*

Lignin and cell‐wall‐bound hydroxycinnamate [*p*coumarate (*p*CA), ferulate (FA) and diferulates (isomer 8–5´l (dfa85l), isomer 8–5´b (dfa85b), isomer 8‐*O*‐4′ (dfa8*O*4) and isomer 5–5′(dfa55)] contents, as well as lignin composition, the percentages of syringyl (S), *p*‐hydroxyphenyl (H) and guaiacyl (G) lignin units, of the stalk pith were evaluated in FS and CS samples using two technical replicates per sample. Three two‐plant samples were used for each treatment‐inbred combination. These analyses allowed us to study the differences between the structural defenses under infestation with *S. nonagrioides* of plants developed from non‐inoculated and inoculated seeds with *F. verticillioides*.

An updated method described by (Santiago et al., 2018) was followed for the extraction of hydroxycinnamates using a sample of 0.5 g of ground tissue. The chromatographic analyses were performed using 2690 Waters HPLC equipment (Waters) with a separation module, a 996 Waters diode detector and an YMC ODS‐AM column (Water; 100 ×3 2 mm i.d.; 3‐μm particle size). The elution conditions were as follows: initial conditions 10:90 (A:B), changing to 30:70 in 3.5 min, 32:68 in 6.5 min, 100:0 in 4 min, then isocratic elution with 100:0 for 4.5 min, and finally returning to initial conditions in 3 min. The flow rate of the mobile phase was 0.3 mL min^−1^, the total analysis time was 21.5 min, and the injection volume of the samples was 4 μL. Quantification was performed at 325 nm. Retention times were compared with *p*‐coumaric and ferulic acid (Sigma) in freshly prepared standard solutions. FA dimers were identified by comparison with the retention time and UV spectra of the 5‐5‐diferulic acid standard and confirmed according to previous literature (Waldron et al., 1996). Calibration curves for the standards were built and used for external quantitation. The DFAT concentration was calculated as the quantified sum of three identified regioisomers, 8‐*O*‐4‐, 5–5‐ and 8–5 (sum of dfa 8‐5‐cyclic form (or benzofuran) and non‐cyclic (linear) form; Ralph et al., 1994).

The lignin content was assessed on a sample of 50 mg using a modification of the Klason lignin quantification protocol of Dence (1992) that has been explained in detail in a previous paper (Gesteiro et al., 2021). Klason lignin contents were expressed as percentages. The lignin composition was determined using oxidation with nitrobenzene as described by Pomar et al., (2002). The HPLC analyses were carried out in the same equipment and under the same conditions described for hydroxycinnamates. The lignin degradation products produced by oxidation with nitrobenzene were quantified at 280 nm using the standards for p‐hydroxybenzaldehyde (H‐subunit indicator), vanillin (G‐subunit indicator) and syringaldehyde (S‐subunit indicator). The values of the subunits were expressed as percentages of the total identified peak areas, and the total amounts were obtained in relation to the polymer quantification determined using the Klason method.

To check the effect of seed colonization by *F. verticilloides* on the maize stem pith nutritional‐value response to the *S. nonagrioides* attack, maize subsamples (1.4–1.6 mg) of the CC, FS and CS treatments were weighed into tin capsules (4 mm Ø x 6 mm) and analysed for elemental composition (% C and % N).

### 2.4 Statistical analyses for developmental traits and untargeted metabolic data

#### 2.4.1 Maize developmental traits

Plant height and stem tunnel length by *S. nonagrioides* feeding were analyzed using the procedure GLM of SAS (SAS 2008). In the linear model, *Fusarium* and *Sesamia* treatments and the level of resistance to FER (resistant versus susceptible) were considered as fixed effects as well as all interactions among those factors. Least square means were compared using the Fisher's protected LSD.

#### 2.4.2 Untargeted metabolic data

As we hypothesize that performance of plants developed from seeds colonized by *F. verticillioides* would differ between resistant and susceptible genotypes to FER and changes on plant height and stem tunneling confirmed that expectation, statistical analyses were independently done for features detected in resistant and susceptible inbreds to FER. To check if plant colonization by *F. verticillioides* could affect the maize response to attack by *S. nonagrioides*, features differentially accumulated (False Discovery Rate (FDR) < 0.10 and Fold Change (FC) >1.5) between CC and CS treatments and between FC and FS were determined using univariate analysis.

As inbred performance against insect attacks of susceptible inbreds seemed to be modified by seed inoculation by *F. verticillioides* and performance of resistant inbreds was not, the features differentially accumulated (FDR <0.10 and FC >1.5) between CS and FS treatments in each group (resistant and susceptible inbreds) were identified and annotated. In addition, a joint analysis with mummichog and a gene set enrichment analysis (GSEA) using the Functional Analysis module of MetaboAnalyst (Pang et al., 2021) was performed to detect metabolic pathway changes between CS and FS treatments. The setup parameters used were: the *Oryza sativa japonica* KEGG pathway library, a mass tolerance of 5 ppm was selected for putative annotation and mummichog default cutoffs for *p*‐values were 0.1 and 0.05 for metabolite data of susceptible and resistant inbreds, respectively.

#### 2.4.3 Cell‐wall‐bound phenolic compounds

We wanted to check the effect of seed infection by *F. verticillioides* on metabolites known to be involved in the defense against *S. nonagrioides* (Gesteiro et al., 2021). Therefore, the analyses of variance for cell‐wall‐bound phenolic compounds were performed on plants infested with *S. nonagrioides* considering all factors as fixed: *Fusarium* treatment (control versus inoculation), the level of resistance to FER (resistant versus susceptible) and the interaction between those factors. Least square means were compared using the Fisher's protected LSD.

#### 2.4.4 Nutritional traits

Initially, exploratory data analyses of %C and %N values were carried out to detect outliers and anomalies that could affect the results, as well as to check the normality of the data distribution (Shapiro–Wilk's W test) and equality of variance among groups (Levene's test). When these assumptions were not fulfilled, the original data were transformed by Tukey's ladder of powers. Then, data were analysed by two‐way ANOVA with treatment as one factor and the level of inbred resistance to *F. verticillioides* as the other factor (resistant versus susceptible). Significant differences were established at *p* < 0.05 using the Bonferroni test for multiple comparisons. The proportion of the variation accounted for each factor or interaction in the ANOVA was determined by the partial eta‐squared η _p_^2^ statistic.

### 2.5 Metabolite annotation

Features were annotated based on their accurate mass, molecular formula, and MS/MS spectrum when available, obtained with the MetaboScape 4.0 software. Putative annotations were performed using publicly available databases as Lipid Maps (https://www.lipidmaps.org), Pubchem (https://pubchem.ncbi.nlm.nih.gov), ChEBI (https://www.ebi.ac.uk/chebi) and Massbank (https://massbank.eu), and consulting literature references.

## 3 RESULTS

**FIGURE 1:**
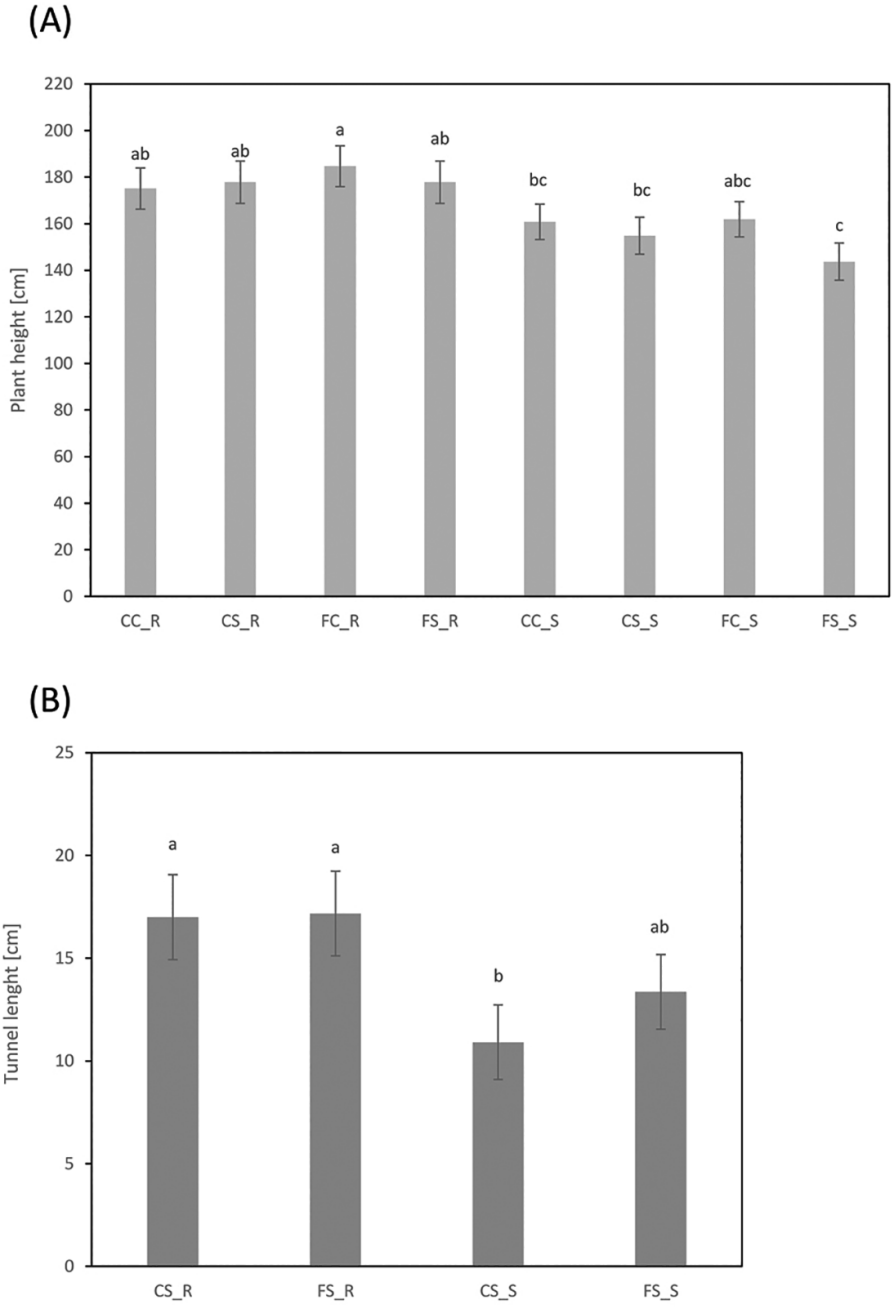
Means of maize inbred lines for developmental and damage traits. Means of resistant (_R) and susceptible (_S) inbreds to Fusarium ear rot (FER) for plant height (A) and stem tunnel length (B) made by *Sesamia nonagrioides*. Inbreds were evaluated under four different treatments: no seed inoculation and no infestation (CC), seed inoculation with *Fusarium* v*erticillioides* and no infestation (FC), no seed inoculation and infestation with *Sesamia nonagrioides* (CS) and seed inoculation with *Fusarium* v*erticillioides* and infestation with *Sesamia nonagrioides* (FS). For each trait, means with the same letter did not significantly differ at *p* < 0.05 (Fisher's Protected LSD).

The effect of kernel infection with *F. verticillioides* was tested on control and *S. nonagrioides* larvae infested plants. Both inbred groups did not differ for plant height and differed for tunnel length at control conditions (CS). They significantly differed for plant height and did not differ for stem tunneling after receiving the double treatment (FS) (Figure 1). Our results have shown that seed infection by *F. verticillioides* differentially affected maize performance under corn borer infestation of resistant and susceptible inbreds to FER. Data for individual inbreds are available in the supplementary table S1.

**FIGURE 2:**
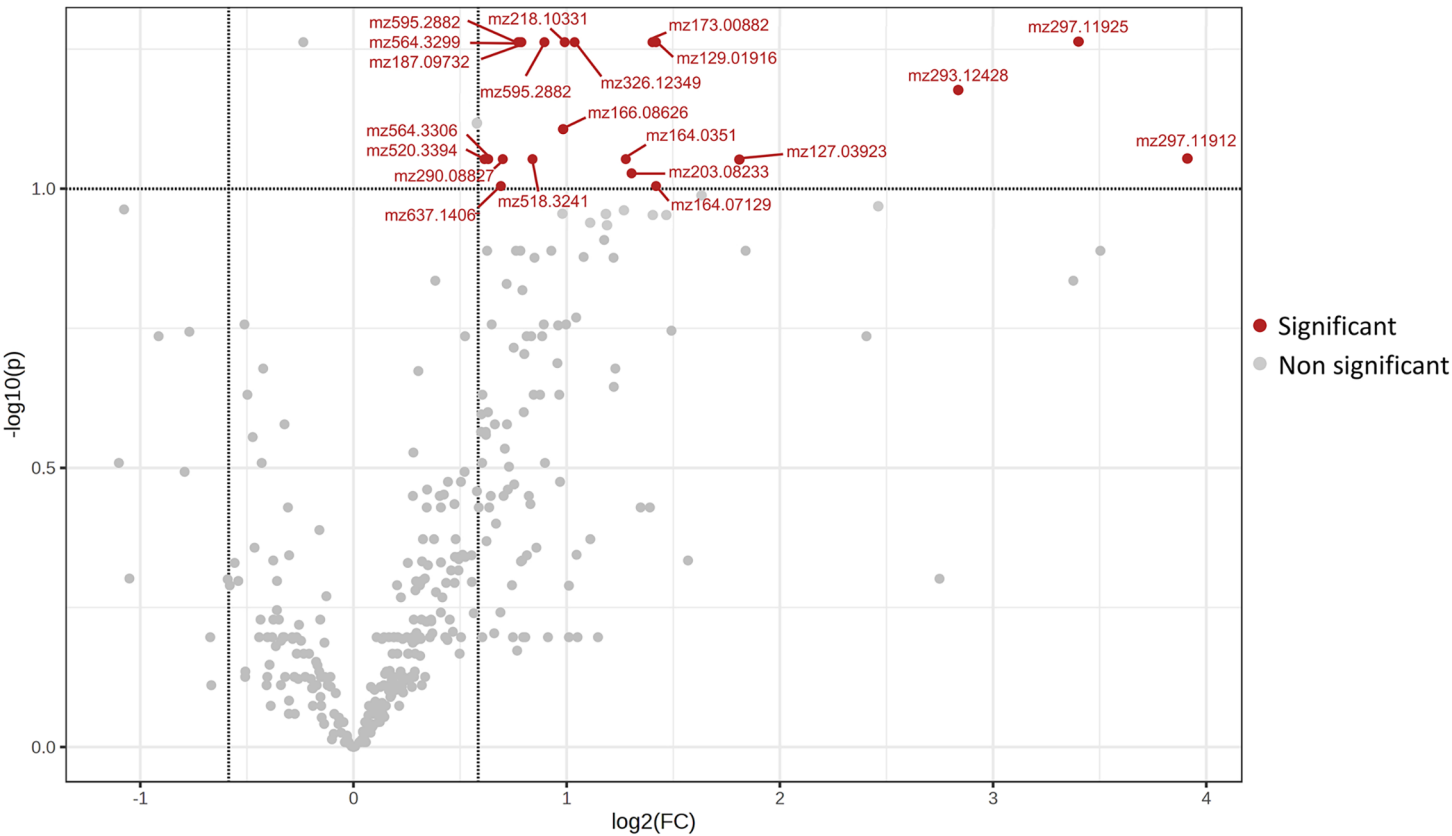
Volcano plot of differentially accumulated compounds between CS and CC treatments in susceptible inbreds. CS stands for infestation with *Sesamia nonagrioides* and CC for control conditions. Features with significant different accumulations (FDR <0.10 and |Log2 Fold Change| > 0.6) between both treatments were highlighted (red dots). Grey dots corresponded to compounds not differentially accumulated.

Univariate analyses detected that 21 compounds were over accumulated (FDR <0.10 and |Log_2_ Fold Change| > 0.6) in the susceptible inbreds under infestation with *Sesamia* (CS) compared to non‐treated plants (CC; Figure 2). Meanwhile, in the resistant inbreds, no features differentially accumulated were found between those treatments (Supplementary figure 1). However, when plants came from seeds inoculated with *F. verticillioides*, no significant changes were observed between control (FC) and infested plants (FS) either for resistant or susceptible inbreds.

**FIGURE 3:**
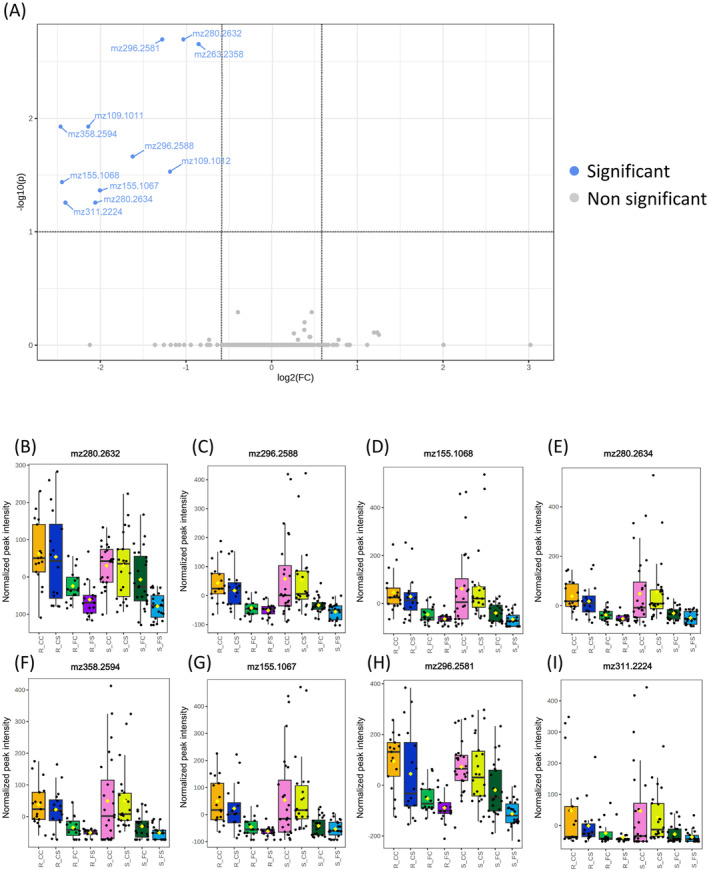
Differentially accumulated compounds in susceptible inbreds between FS and CS treatments. FS stands for the double treatment and CS for the infestation treatment with no infection. (A) Volcano plot in which features with FDR <0.10 and |Log2 Fold Change| > 0.6 were highlighted (blue dots) as differentially accumulated. Grey dots corresponded to compounds not differentially accumulated. (B) Box plots of data distribution of resistant (R_) and susceptible (S_) inbreds under each treatment for the compound mz280.26332. The different treatments are: no seed inoculation and no infestation (CC), seed inoculation with *Fusarium* v*erticillioides* and no infestation (FC), no seed inoculation and infestation with *Sesamia nonagrioides* (CS) and seed inoculation with *Fusarium* v*erticillioides* and infestation with *Sesamia nonagrioides* (FS). The box upper and lower limits represent the third and first quartiles, respectively. The intermediate line stands for the median value and whiskers delimit the range of typical values. (C) Box plots of data distribution for the compound mz296.2588, (D) mz155.1068, (E) mz280.2634, (F) mz358.2594, (G) mz155.1067, (H) 296.2581, and (I) mz311.2224.

In the susceptible inbreds, 11 features were differentially accumulated between CS and FS treatments (Figure 3) and were putatively identified as eight metabolites (Table 2). Meanwhile no metabolites were differentially accumulated in the resistant inbreds (Supplementary figure 2). Putative annotations were based on comparison of analytes accurate mass, molecular formula, and MS/MS spectrum with public databases data, literature references, and analytical standards when necessary. The metabolites annotated are lipids or lipid related analytes, such as a hydroperoxylinoleic acid (HPODE) and hydroxydodecanoylcarnitine (Table 2). Protonated ions at m/z 280.2 were tentatively annotated as monounsaturated primary fatty acids amides as their fragmentation patterns show the characteristic [M + H‐17]^+^ and [M + H‐35]^+^ product ions from the loss of ammonia and water molecules (Divito et al., 2012). Based on standard analysis, the primary fatty acid linoleamide, with equivalent molecular formula, mass, and fragmentation pattern (Li et al., 2023) was discarded. Compounds with a protonated ion at m/z 296.2 are listed as linolenic acid derivatives based on characteristics ions at m/z 279.2, 261.2 and 243.2 in their fragmentation patterns (Geng et al., 2015). Analytes left unidentified and not included in Table 2, such as protonated ions at m/z 109.1 and m/z 263.2, could be produced by in‐source fragmentation during electrospray ionization, since they coeluted with other compounds and are coincident with ions from their fragmentation patterns.

**TABLE 2:** Features differentially accumulated in susceptible inbreds to *Fusarium verticillioides* between Control‐*Sesamia* (CS) and *Fusarium*‐*Sesamia* (FS) treatments.

Feature identification	RT (min)	Neutral mass	m/z	Ionization	MS/MS fragments (m/z)	Molecular formula	Error (|Δ m/z| ppm)	Tentative compound		log_2_FC	*p*
**mz280.2632**	24.85	279.2559	280.2632	[M + H]+	263.225, 245.214, 221.21, 179.167, 165.153, 151.138, 147.109, 133.093, 123.108, 114.082, 109.093, 95.078, 81.063, 67.048, 55.049		C18H33NO	1.19	Monounsaturated primary fatty acid amide (Divito et al., 2012)	−1.2775	0.0020006
**mz296.2588**	25.13	295.2515	296.2588	[M + H]+	279.231, 261.227, 251.229, 243.210, 234.228, 221.115, 219.171, 165.136, 147.118, 141.121, 135.113, 109.103, 99.081, 95.087, 81.071, 67.055, 55.055		C18H33NO2	1.01	Linolenic acid derivative (Geng et al., 2015)	−0.85221	0.0022073
**mz155.1068**	25.13	154.0995	155.1068	[M + H]+	140.994, 109.095, 105.040, 95.045, 77.036, 53.037, 51.021		C9H14O2	0.65	Unknown	−2.4636	0.011808
**mz280.2634**	25.16	279.2561	280.2634	[M + H]+	263.225, 245.214, 221.21, 179.167, 175.137, 165.153, 151.138, 147.107, 133.092, 123.092, 114.083, 109.093, 95.078, 81.063, 67.048, 55.049		C18H33NO	0.25	Monounsaturated primary fatty acid amide (Divito et al., 2012)	−1.6209	0.021799
**mz358.2594**	25.20	359.2667	358.2594	[M‐H]‐	354.205, 309.319, 293.179, 288.030,277.218, 205.097, 162.073		C19H37NO5	1.27	Hydroxydodecanoylcarnitine	−1.1905	0.029381
**mz155.1067**	25.23	154.0995	155.1067	[M + H]+	‐		C9H14O2	0.74	Unknown	−2.448	0.036452
**mz296.2581**	25.32	295.2509	296.2581	[M + H]+	279.218, 261.211, 183.130, 153.119, 141.120, 109.095, 95.082, 81.065, 67.051, 55.052		C18H33NO2	0.65	Linolenic acid derivative (Geng et al., 2015)	−2.4054	0.055289
**mz311.2224**	25.62	312.2297	311.2224	[M‐H]‐	309.173, 299.024, 293.198, 223.135, 183.007, 125.0103, 113.092		C18H3204	1.30	Hydroperoxylinoleic acid (Li et al., 2022)	−2.0557	0.055289

Notes: RT, Retention time (minutes); m/z, mass number/charge number; Log_2_FC, Log_2_ Fold change between FS and CS treatments; *p*, *p*‐adjusted value.

**FIGURE 4:**
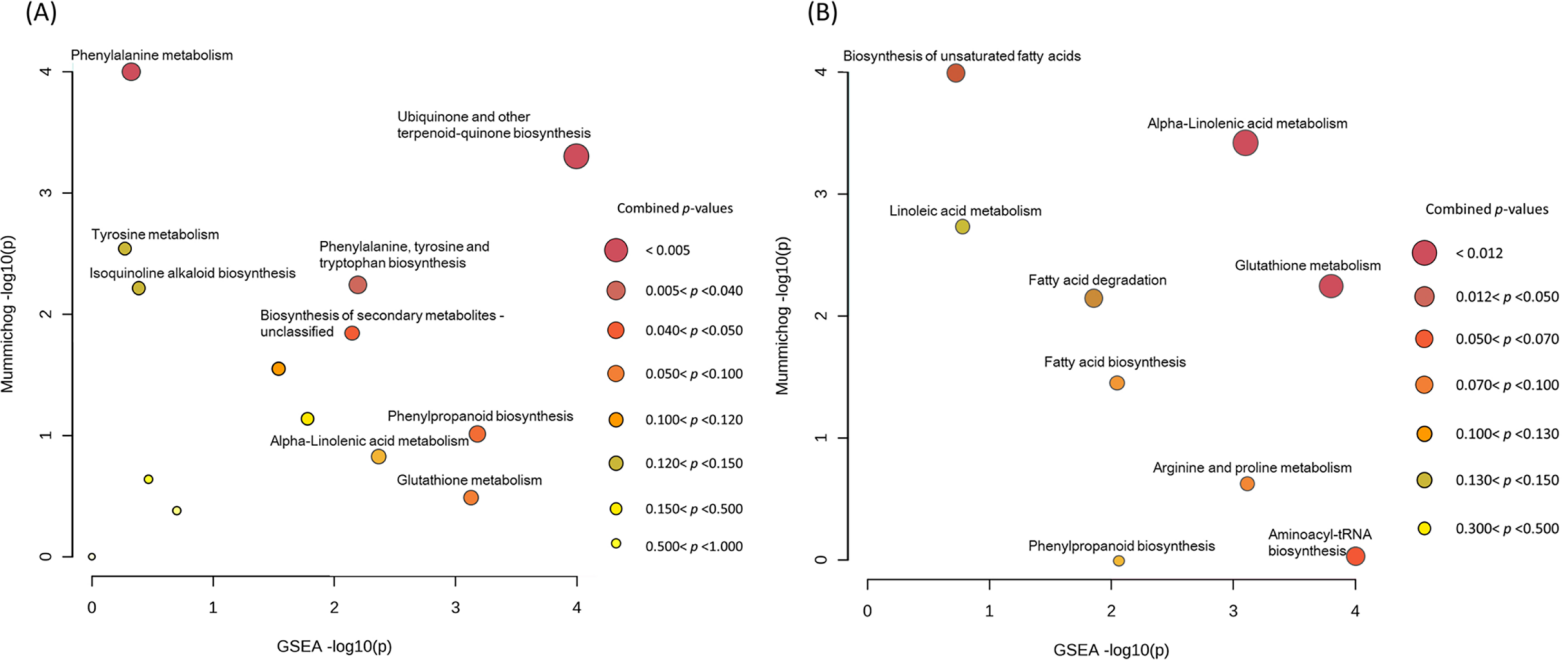
Functional analysis of differential accumulation between FS and CS. FS stands for seed inoculation with *F. verticillioides* and infestation with *S. nonagrioides* and CS for no seed inoculation with *F. verticillioides* and infestation with *S. nonagrioides* treatments. Functional analysis was separately done in the stem pith samples of resistant (A) and susceptible (B) inbreds to FER. Functional analysis uses the “mummichog” algorithm in combination with a set renricment analysis (GSEA) to predict pathway activities (Pang et al., 2021). Larger and more reddish circles (smaller combined *p*‐value) represent more reliably perturbed pathways.

The Functional Analysis results showed that, in general, pathways most enriched among metabolites differentially accumulated between CS and FS in resistant inbreds (Figure 4 A) differed from those differentially accumulated in susceptible inbreds (Figure 4 B). Under infestation with *S. nonagrioides*, the biosynthesis of unsaturated fatty acids and alpha‐linolenic acid metabolism would be downregulated and glutathione metabolism and aminoacyl‐tRNA biosynthesis would be upregulated by seed infection in the susceptible plants. On the other hand, the phenylalanine, tyrosine and tryptophan biosynthesis (PTTB) pathway was enriched in the comparison FS versus CS in resistant inbreds, as well as other pathways highly related with the PTTB pathway such as the isoquinoline alkaloid biosynthesis and the ubiquinone and other‐terpenoid‐quinone biosynthesis pathways.

The analyses of variance for cell‐wall‐bound phenolic compounds showed significant differences between resistant and susceptible inbreds to infection by *F. verticillioides* for *p*CA, FA, dfa85l, dfa85b, dfa55, and DFAT. Differences between FS and CS treatments were significant for FA, dfa8o4, dfa55, dfa85b, and DFAT. Finally, the resistance group x *Fusarium* treatment interaction was significant for the percentage of syringyl lignin units and dfa85l content (data not shown). The mean comparisons for cell‐wall‐bound phenolic compounds are shown in Table 3. Seed inoculation with *F. verticillioides*, upon infestation with *S. nonagrioides*, significantly increased the amount of ferulate and diferulates in the stem pith and, in general, resistant inbreds to *F. verticillioides* showed higher levels for hydroxycinnamates than susceptible inbreds. However, changes driven by seed inoculation with *F. verticillioides* on the percentage of syringyl lignin units were dependent on the level of resistance of the inbreds. In general, among resistant inbreds, S was higher under FS than under CS treatments, but the opposite was true among susceptible inbreds (Table 3 and Supplementary Table S1).

**TABLE 3:** Least square means for cell‐wall‐bound phenolic compound (lignin and hydroxycinnamates) contents and lignin composition in the stem pith of inbreds resistant and susceptible to *Fusarium verticillioides* evaluated under infestation with *Sesamia nonagrioides* and two inoculation treatments (seed inoculated with *Fusarium verticillioides* (FS) and control (CS)).

Resistance	Treatment	KL (%) ^1^	S (%)	G (%)	H (%)	PCA (mg/g)	FA (mg/g)	dfa85l (mg/g)	Dfa85b (mg/g)	dfa8o4 (mg/g)	dfa55 (mg/g)	DFAT (mg/g)
Resistant	FS	15.9 a	47.7 a	40.5 a	11.8 a	7.7 a	2.2 a	0.07 b	0.18 a	0.22 a	0.10 a	0.57 a
Resistant	CS	14.4 a	45.5 ab	41.3 a	13.5 a	7.0 ab	1.7 bc	0.09 a	0.12 b	0.12 b	0.08 b	0.41 b
Susceptible	FS	18.0 a	44.4 b	43.3 a	12.2 a	5.7 ab	1.8 ab	0.06 b	0.15 ab	0.22 a	0.08 b	0.51 ab
Susceptible	CS	16.3 a	46.9 ab	41.1 a	12.0 a	4.7 b	1.3 c	0.05 b	0.07 c	0.08 b	0.05 c	0.25 c

Within each column, means followed by the same letter did not significantly differ at 0.05 probability level.

Notes: ^1^KL = Kason lignin content; S = Percentage of syringyl lignin units; G = Percentage of *p*‐hydroxyphenyl lignin units, H = Percentage of guaiacyl units; PCA = Cell‐wall‐bound *p*‐coumarate content; FA = Cell‐wall‐bound Ferulate content; dfa85l = Diferulate isomer 8–5´l (linear form); dfa85b = Diferulate isomer 8–5´b (benzofuram form); dfa8o4 = Diferulate isomer 8‐o‐4′; dfa55 = Diferulate isomer 5–5′ and DFAT = Total diferulate content.

**FIGURE 5:**
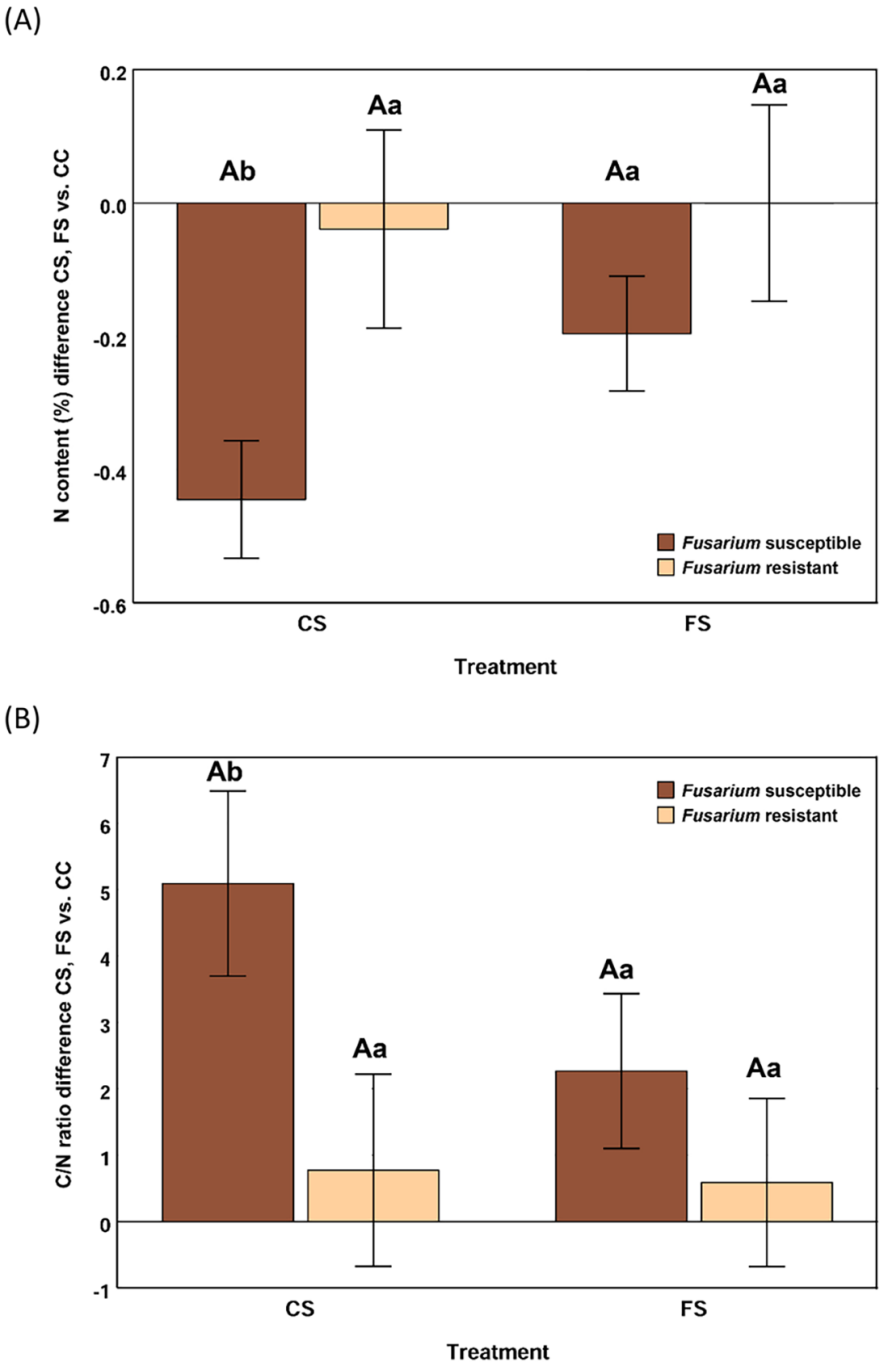
*C*/N and N content responses to attack by *Sesamia nonagrioides*. Comparison between responses to *Sesamia* infestation of resistant (light boxes) and susceptible (dark boxes) inbreds to *Fusarium verticillioides* for *C*/N and N contents in the maize stem pith. Those responses were measured under two different conditions: infestation with *Sesamia nonagrioides* alone (CS) or in combination with seed inoculation with *Fusarium verticillioides* (FS). For each treatment, responses of resistant and susceptible inbreds with the same lowercase letter did not significantly differ at 0.05 probability level. Meanwhile, within each inbred group, responses to FS and CS with the same uppercase letter did not significantly differ.

The analyses of variance showed that responses of resistant and susceptible inbreds to infestation treatments (CS and FS) significantly differed for the C/N ratio (*p* = 0.028) and N content (*p* = 0.011) of the stem pith. Upon infestation with *S. nonagrioides* alone, a reduction of N content and an increase of C/N was induced in the susceptible inbreds. Meanwhile, changes in the resistant inbreds were small, resulting in significant differences between the responses of resistant and susceptible inbreds (Figure 5). However, the presence of *F. verticillioides* moderated the induced response by *S. nonagrioides* of the susceptible inbreds and the responses of resistant and susceptible inbreds did no longer differ.

## 4 DISCUSSION

As *F. verticillioides* can function as a pathogen or an endophyte, the effect of seed infection by *F. verticillioides* on maize performance has been independently studied in genotypes that showed resistance and susceptibility to FER. Our hypothesis was that, in the resistant genotypes, seed infection could have some beneficial consequence on maize development and/or resistance to other microorganisms, meanwhile the effect could be detrimental in susceptible inbreds. Among resistant inbreds, although plant height tended to increase in plants developed from inoculated seeds compared to *Fusarium*‐free seeds, differences among treatments were not significant and, consequently, an endophytic interaction between the fungus and the plant has not been proven to happen in those genotypes. However, differences of performance against insect attack between resistant and susceptible genotypes were conditioned by seed treatment. Subtle changes promoted by fungal infection being unfavorable for the susceptible inbreds suggesting that, in these genotypes both biotic factors, kernel infection by *F. verticillioides* and insect attack, could interact in a synergistic way resulting in increased vulnerability of the maize plant (Caesar 2003). Schulthess et al., (2002) have already proposed that maize systemic fungal infection by *F. verticillioides* could interfere in some way with host plant insect resistance mechanisms. In addition, the un‐targeted metabolic response of susceptible inbreds to *Sesamia* attack was mitigated in plants developed from infected seeds, suggesting that seed infection could make susceptible genotypes more vulnerable to the stress caused by insect attacks. Agreeing with this hypothesis, N depletion and C/N enrichment caused by maize attack by *S. nonagrioides* on plants developed from *Fusarium*‐free seeds was significantly higher in the susceptible compared to resistant inbreds. However, the differences between resistant and susceptible inbreds were not significant among plants from seeds infected with *F. verticillioides* N content depletion and C/N enrichment being associated to induced defense against insect attack (Cornelissen and Fernandes 2001; Rasmann et al., 2009; Liu et al., 2010; Rothhaupt et al., 2015; Fotelli et al., 2020). Fernández‐Descalzo et al., (2024) also reported a significant decrease of δ^13^C in FS plants compared to CC plants, around 0.3‰, but only half the decrease recorded in CS plants. They suggested that seed infection by *Fusarium* could reduce the plant capacity to regulate defence and carbon assimilation pathways in response to insect attacks (Meza‐Canales et al., 2017). Overall, the relationship between *S. nonagrioides*, *F. verticillioides* and maize is more complex than expected from previous studies in which ear damage by *S. nonagrioides* was established as one of the most influential factors on FER and kernel contamination with fumonisins because seed infection by *F. verticillioides* could also modify plant defenses against insect attacks (Blandino et al., 2008; Santiago et al., 2015). Therefore, in field conditions, seed inoculation cannot be recommended as a favorable treatment for maize cultivation because the modification of plant defenses against insect attack by *F. verticillioides* seed inoculation could increase the susceptibility in some genotypes and no significant favorable effect on maize development was observed.

Consequently, features differentially accumulated between the double treatment (FS) and the *Sesamia* treatment alone (CS) could have an important role on the modification of performance of susceptible inbreds under *Sesamia* attack. Most of the differentially accumulated metabolites between FS and CS treatments were lipids or lipid‐derived compounds. In addition, we searched for metabolic pathways that could be differentially regulated in *Sesamia*‐infested plants coming from *Fusarium*‐infected and *Fusarium*‐free seeds. The biosynthesis of unsaturated fatty acids and linolenic acid metabolism seemed to be downregulated in susceptible inbreds under the double treatment compared to *Sesamia* treatment alone. Therefore, in susceptible inbreds, the lipid signature of seed infection by *F. verticillioides* can be involved in the worsening of maize performance against *S. nonagrioides* attacks. Previous studies had already pointed out that the maize lipidome signature was genotype‐dependent and was strongly involved in the maize‐*F. verticillioides* interaction, but results from this study go further as they show that the fungus‐plant genotype interaction can also affect maize defense behavior against other parasites (Righetti et al., 2019; Cao et al., 2023).

Metabolites less accumulated in susceptible inbreds under FS treatment than under CS treatment were putatively annotated as two monounsaturated primary fatty acid amides, two linolenic acid derivatives, hydroperoxylinoleic acid, and hydroxydecanoylcarnitine. Hydroxydecanoylcarnitine is an acylcarnitine and, in plants, acylcarnitines are associated with processes of lipid metabolism, in anabolic and possibly in catabolic processes (Nguyen et al., 2016). Similarly, it is well known that compounds derived from the octodecanoid pathway, as linoleic acid derivatives, activate defensive genes that initiate “induced systemic resistance” against insect herbivores (Walling 2000). Therefore, lipid responses to seed infections by *F. verticillioides* could mediate the increased plant susceptibility to insect attack by interfering in the plant response to herbivory.

In the resistant inbreds, isoquinoline alkaloid biosynthesis and ubiquinone and other‐terpenoid‐quinone biosynthesis pathways were enriched among metabolites differentially expressed under FS and CS treatments in agreement with previous studies (Cao et al., 2022; Cao et al., 2023). Cao et al., (2022) revealed higher expression of genes involved in electron transport, ubiquinone being a key component of the electron transport chain, in resistant compared to susceptible maize recombinant inbred lines (RILs) as well as lower expression of genes involved in the tricarboxylic acid cycle and suggested it could be due to a higher activation of amino acid synthesis in resistant versus susceptible RILs. Cao et al., (2022) and other authors (Wang et al., 2016; Ge et al., 2021) have also shown significantly higher accumulation of isoquinoline and an increased expression of the gene *S‐norcoclaurine synthase 1* among resistant inbreds compared to susceptible inbreds. The *S‐norcoclaurine synthase 1* gene catalyzes the first step of the isoquinoline alkaloids biosynthesis. The isoquinoline alkaloids have been proposed as antifungal compounds and specifically show an inhibitory action against fumonisin biosynthesis (Tims and Batista 2007, Wong‐Deyrup et al., 2021). On the other hand, ubiquinone is constituted of a benzene quinone ring precursor synthesized from tyrosine or phenylalanine (Diamond and Desgagne‐Penix 2016; Liu and Lu 2016). In the current study, metabolites of the phenylalanine, tyrosine and tryptophan biosynthesis and metabolism pathways were also overrepresented in the comparison FS versus CS in resistant inbreds in accordance with results obtained by Ciasca et al., (2020). These authors indicated that tyrosine was significantly more accumulated in an inbred line resistant to *F. verticillioides* infection than in its susceptible counterpart, while the opposite was true for L‐tryptophan. Isoquinoline alkaloid biosynthesis, phenylalanine metabolism, tyrosine biosynthesis, and tryptophan biosynthesis were also enriched in the response of *Vicia faba* to the treatment with a *Bacillus velezensis* peptide that exhibits good field control of Fusarium wilt caused by *Fusarium oxysporum* (Zhang et al., 2022). Therefore, phenylalanine, tyrosine and tryptophan biosynthesis and metabolism pathways related compounds could be crucial to establish a favorable interaction between the host (maize) and the fungus (*F. verticillioides*) to fight against *S. nonagrioides* attacks.

In the current study, accumulation of compounds of the phenylpropanoid pathway such as the cell‐wall‐bound hydroxycinnamates was induced by seed inoculation with *F. verticillioides* upon infestation with *S. nonagrioides*, independently of the level of maize inbred resistance to *Fusarium*, but the percentage of syringyl lignin units was increased in resistant inbreds and reduced in susceptible inbreds. High S subunit‐enriched lignin content has been associated with increased resistance to stem tunneling by *S. nonagrioides* (Gesteiro et al., 2021) and to other biotic stresses (Wuyts et al., 2006, Eynck et al., 2012, Zheng et al., 2017, Ma et al., 2018). Therefore, differential changes in lignin composition primed by seed infection by *F. verticillioides*, upon plant infestation, between resistant and susceptible inbreds to FER could contribute to reduced defense differences between both inbred groups.

As conclusions, an endophytic interaction between the fungus and some plant genotypes cannot be unquestionable proven. However, exploration of maize metabolism differentially primed by *F. verticillioides* seed infections upon maize infestation with *S. nonagrioides*, has shown that defense metabolites and pathways were favorably altered among resistant inbreds to *F. verticillioides* and were not among susceptible inbreds.

## AUTHOR CONTRIBUTIONS

A.B. conceived the study and took care of the greenhouse experiment, data recording, and sample collection with the assistance of R.S., A.C., N.G. and R.A.M.. A.C. led extraction, acquisition and filtering of UHPLC‐QTOF data with the assistance of N.G., A.C. performed the identification of tentative compounds, R.S. led extraction and quantification by HPLC of cell‐wall components with the assistance of P.L., S.G.P. performed the C and N elemental analyses, A.B., A.C. and N.G. performed the statistical analyses of data. A.B. and A.C. drafted the initial manuscript and N.G. edited the figures. All authors have read and approved the final version of the manuscript.

## FUNDING INFORMATION

This research was funded by subsequent coordinated projects financed by MCIU/AEI/FEDER, UE (RTI2018‐096776‐B‐C21, RTI2018‐096776‐B‐C22, PID2021‐122196OB‐C21 and PID2021‐122196OB‐C22).

## CONFLICT OF INTEREST STATEMENT

No conflict of interest declared.

## Supporting information


Figure S1.

Figure S2.

Table S1.

## Data Availability

Raw metabolic data are deposited in DIGITAL.CSIC (https://doi.org/10.20350/digitalCSIC/16601) and freely available under CC‐BY license.
