# Supplementary figures and images for: Effects of seed infection by Fusarium verticillioides on maize performance against Sesamia nonagrioides attack
Source: Physiol Plant. 2024 Dec 3;176(6):e14649. doi: 10.1111/ppl.14649 (PMC11615131; doi:10.1111/ppl.14649)

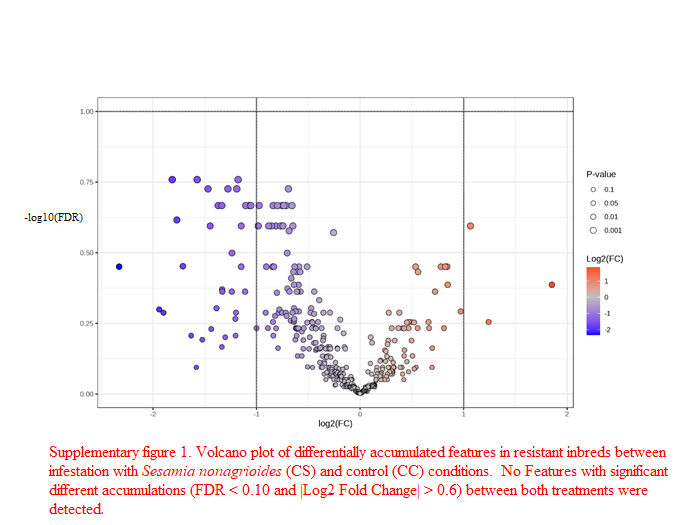

Supplement: Supplementary file 1 — Figure S1. [file PPL-176-e14649-s003.tif]

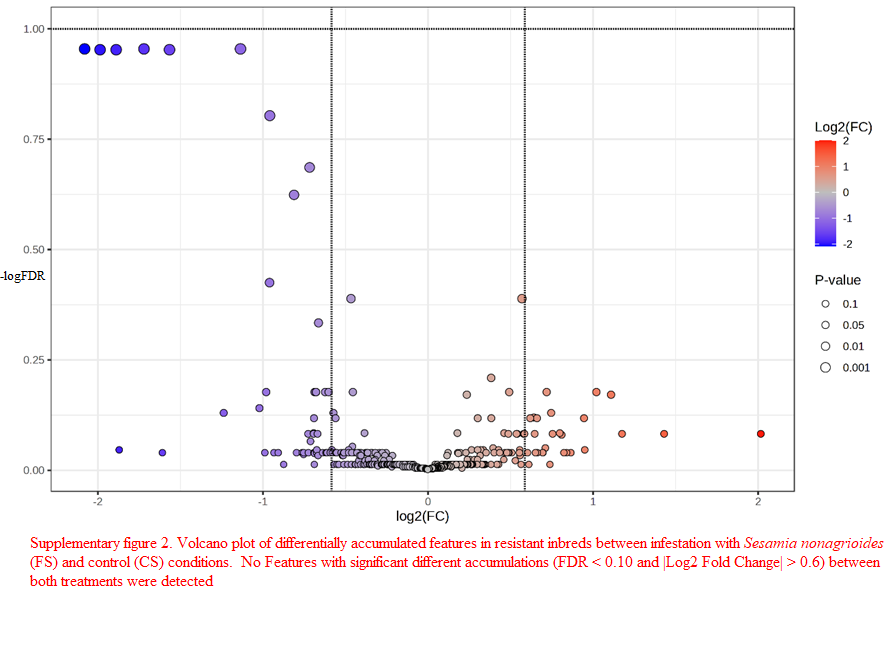

Supplement: Supplementary file 2 — Figure S2. [file PPL-176-e14649-s001.tif]
